# Foraging bee species differentially prioritize quantity and quality of floral rewards

**DOI:** 10.1093/pnasnexus/pgae443

**Published:** 2024-10-07

**Authors:** Jaya Sravanthi Mokkapati, Michael Hill, Natalie K Boyle, Pierre Ouvrard, Adrien Sicard, Christina M Grozinger

**Affiliations:** Department of Entomology, Center for Pollinator Research, Huck Institutes of the Life Sciences, Pennsylvania State University, University Park, PA 16802, USA; Department of Entomology, Center for Pollinator Research, Huck Institutes of the Life Sciences, Pennsylvania State University, University Park, PA 16802, USA; Department of Entomology, Center for Pollinator Research, Huck Institutes of the Life Sciences, Pennsylvania State University, University Park, PA 16802, USA; Department of Plant Biology, Uppsala Biocenter, BOX 7080, 750 07 Uppsala, Sweden; Department of Plant Biology, Uppsala Biocenter, BOX 7080, 750 07 Uppsala, Sweden; Department of Entomology, Center for Pollinator Research, Huck Institutes of the Life Sciences, Pennsylvania State University, University Park, PA 16802, USA

**Keywords:** bee foraging preferences, plant–pollinator interactions, pollination ecology, floral traits, nutritional ecology

## Abstract

Pollinator–plant interactions represent a core mutualism that underpins biodiversity in terrestrial ecosystems, and the loss of flowering plants is a major driver of pollinator declines. Bee attraction to flowers is mediated by both quantity of resources (the number of available flowers for exploration) and quality of resources (pollen nutritional value), but whether and how bees prioritize these factors is not well understood. Here, we leveraged a unique plant system to investigate the floral factors influencing bee foraging decisions. Recombinant inbred plant lines were generated by crossing the self-fertilizing *Capsella rubella* and the pollinator-dependent outcrosser *C. grandiflora*, to produce plants that varied across floral traits. Using enclosed arenas, we evaluated the foraging behavior of two solitary bee species, *Osmia cornifrons* and *Megachile rotundata*, to the isolated inflorescences from these lines. Visits from *O. cornifrons* were significantly positively correlated with the number of flowers, while *M. rotundata* visits were significantly positively associated with pollen nutrition, with a preference for plants with higher pollen protein-to-lipid content. Further experiments using artificial flowers confirmed that *M. rotundata* preferred flowers with higher protein:lipid ratios, while *O. cornifrons* visits were unaffected by nutrition. These studies demonstrate that, although both bee species collect pollen as their sole source of protein and lipids for themselves and/or their offspring, they differentially prioritize resource quantity (number of flowers) and quality (pollen nutritional content). These studies lay the groundwork for understanding how different foraging strategies evolved, and influence, plant–pollinator ecological networks.

**Significance Statement**

Global declines of pollinator populations pose significant concerns for crop production and biodiversity. Understanding the nutritional needs of different pollinator species and how they shape foraging preferences is essential for creating and managing habitat to support and conserve pollinators. Here, we demonstrate that one solitary bee species selects flowering plants based on the number of flowers, prioritizing resource quantity, while another solitary bee species preferentially chooses plants based on the nutritional quality (protein and lipid ratio) of the pollen. Thus, bee species prioritize different aspects of floral resources when foraging. Information on the foraging strategies of different bee species is essential for understanding how plant–pollinator interactions evolved and for managing landscapes to support pollinators and pollination services.

## Introduction

Bees and other animals play a crucial role in pollinating many flowering plants (1), including agricultural crops (2), and their decline could have far-reaching consequences for both natural ecosystems and agricultural production. Most bee species rely on flowering plants for nectar and pollen, which provide them with essential nutrients (3). When their foraging options are limited to a narrow range of plant species, such as in monoculture farming systems, bees may not be able to meet their nutritional needs, which can impact their overall health and fitness (4, 5). Thus, there is increasing interest in developing cost-effective habitat management and restoration schemes that can improve nutritional availability for diverse pollinators (6, 7).

The mutualistic relationship between flowering plants and their insect pollinators has led to the evolution of a diverse array of plants and pollinators with different traits and complex relationships (8). Because plant and pollinator traits have co-evolved and been shaped by the ecological community context, it is difficult to identify which traits are primary drivers of visitation patterns, and thus whether different pollinator species prioritize different floral traits, in natural co-evolved plant–pollinator systems. Understanding the nutritional and foraging preferences of diverse bee species is essential for designing habitats that can support targeted bee species and/or diverse pollinator communities (7).

Most bee species depend on pollen collected from flowers for their main source of proteins and lipids, as well as several micronutrients such as vitamins and minerals (3). Protein and lipids from pollen are essential for reproduction, immune function, energy (9–11) in bees, and to feed their developing brood (7). Different plant species produce pollen with varying concentrations of proteins and lipids (between 2 and 60% for proteins and 1 to 20% for lipids) (12–14). Vaudo et al. (15). demonstrated that co-foraging bees in a natural ecological community partition the plant community into nutritional niches, foraging to obtain pollen with different protein-to-lipid (P:L) ratios. Detailed studies on *Bombus impatiens* and *Bombus terrestris* bumble bees demonstrated that they preferentially forage to collect pollen with a specific P:L ratio (16, 17). However, in these studies foraging rates were normalized to floral area. Indeed, several studies have demonstrated that bees preferentially forage on plants with more flowers or larger flowers (thus, larger floral display areas) (18–20). Thus, the extent to which bees prioritize floral resource quanity (floral area) vs. floral resource nutritional quality remains to be determined.

Plants belonging to Brassicaceae family attract several types of pollinators including *Osmia* mason bees (21) and *Megachile* leafcutting bees (22). The plant genus *Capsella* (family: Brassicaceae) provides a unique opportunity in which to uncouple floral traits and evaluate bee foraging preferences. The predominantly self-fertilizing *C. rubella* (*Cr*) has evolved from the outbreeding and pollinator-dependent ancestral species *C. grandiflora* (*Cg*) via mutations inactivating the self-incompatibility locus which prevents self-pollination (23). The transition from outbreeding (as in *Cg*) to self-compatibility (as in *Cr*) is a common phenomenon in plants and, as is the case in *Cr*, is often accompanied by changes in floral traits that are involved in pollinator reward and attraction (24). These changes can include reduced flower size, modifications in flower shape or structure, as well as reduced production of pollen and nectar, and/or volatile organic compounds (25, 26). Crossing *Cr* and *Cg* to create different recombinant inbred lines (RILs) is thus a powerful approach for uncoupling different floral traits that attract and reward pollinators, to better understand the relative importance of these different traits to different pollinator species, and how these preferences shape the evolution of plant–pollinator networks (27).

**Fig. 1.**
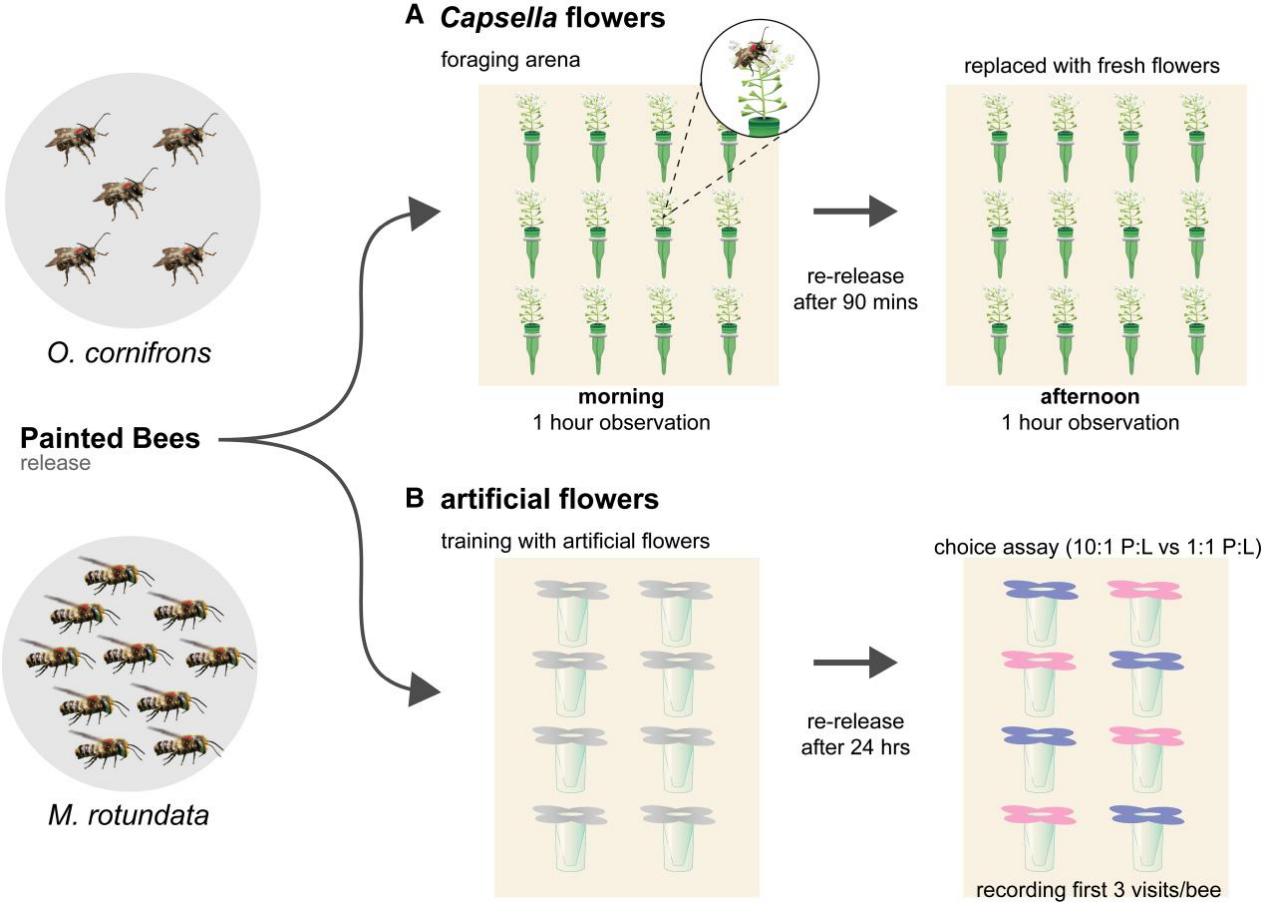
Schematic representation of foraging trials using two bee species. *Osmia cornifrons* and *M. rotundata* were assayed in indoor foraging arenas using A) *Capsella* flowers—in each trial, the arena was equipped with 12 different inflorescences from 10 *Capsella* recombinant plant lines and their 2 parents (*Cg* and *Cr*). A total of 20 trials (10 trials per group of plant lines) per bee species were conducted using 5 bees/trial for *O. cornifrons* and 10 bees/trial for *M. rotundata*, and B) artificial feeders—in each trial, 4 diets each of either 10:1 or 1:1 P:L (protein-to-lipid ratio) were randomly placed in the arena. A total of 15 trials were performed for each species using 5 bees/trial. Each bee was paint-marked for individual identification during observations. Individual visits and their duration were recorded for each bee during each trial. Graphic design by Michael Tribone (Penn State University).

In this study, we evaluated the foraging preferences of two solitary bee species, *Osmia cornifrons* (horned-faced mason bees) and *Megachile rotundata* (alfalfa leafcutting bees) using 20 *Capsella* RILs (intercrossed populations of *Cg* and *Cr*, Sicard et al. (28)) in a lab-based foraging assay (see Fig. 1). We aimed to understand (ⅰ) whether bees exhibit species-specific attraction for different floral traits i.e. floral resource quantity (here evaluated as the number and size of flowers) and/or floral nutritional quality (in terms of pollen protein concentration, lipid concentration, and P:L ratios) and (ⅱ) the relationship between the suite of floral traits of *Capsella* inbred lines and the cumulative visitation for each bee species. To validate the results of these experiments, we assessed whether the foraging choices of these two bee species differ exclusively based on specific P:L ratios in food using artificial flowers in a laboratory choice test.

## Results

### Floral traits vary among recombinant inbred *Capsella* plant lines

**Fig. 2.**
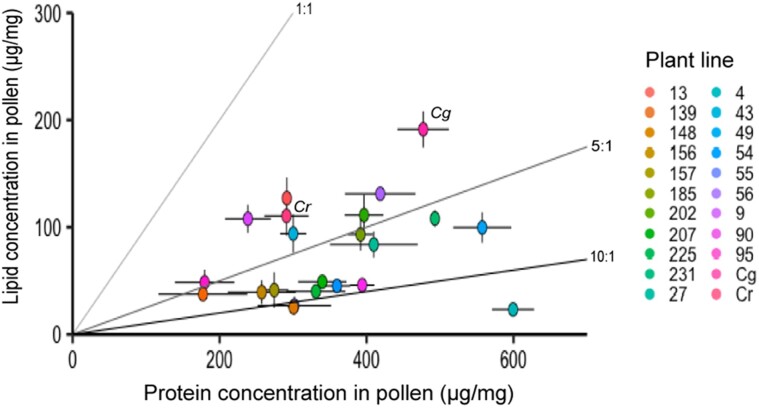
Macronutrient composition across different *Capsella* recombinant inbred plant lines produced by crossing pollinator-dependent *Cg* and self-reproducing *Cr*. Solid rails emphasize the plant lines that lie within 10:1–5:1 and 5:1–1:1 protein:lipid (P:L) range. Error bars indicate standard error of mean for each plant line.

Floral traits for 20 *Capsella* RILs and the two parental lines (*Cg* and *Cr*) varied substantially (see Table 1). Average petal sizes varied across all RILs in the range between those of parental plant lines (highest petal area/width for *Cg* and lowest for *Cr*) (note that RIL 185 has the highest average petal length) (Table 1). The total quantities of proteins and lipids in dried pollen varied across all RILs (depicted in Fig. 2). The P:L values across RILs ranged from 2.21:1 (for RIL 009) to 25.78:1 (for RIL 004), but most RILs exhibited P:L ratios less than 10:1 (only RIL 004, RIL 055, and RIL 148 have P:L ratios over 10:1). Although the values of P:L for *Capsella* parental lines appeared to be similar (2.49:1 for *Cg* vs. 2.63:1 for *Cr*), the pollinator-dependent parental line *Cg* contained almost double (1.8 times) quantities of total proteins and lipids in pollen than the selfing *Cr* plant line (Table 1). Moreover, in terms of quantities, some RILs contain either proteins or lipids beyond the range of values present in their parental lines (Table 1). Among all *Capsella* plant lines used for this study, RIL 004 had the greatest protein concentration in pollen (mean ± SE 599.3.9 ± 28.6 µg/mg pollen), and the fewest total lipids (23.2 ± 3.3 µg/mg pollen). In contrast, RIL 139 had the least quantity of total proteins (177.3 ± 60.5 µg/mg pollen) compared to other plant lines.

**Table 1.** Morphological and nutritional characteristics of the *Capsella* parental plant lines (*Cg* and *Cr*) and their RILs used for the study.

Plant line	Type	Group^a^	Floral traits			Macronutrient concentrations		
			Petal area (mm^2^)	Petal length (mm)	Petal width (mm)	Total proteins (µg/mg)	Total lipids (µg/mg)	Protein:lipid
*Cg*	Parent	I and II	15.2 ± 3.2	5.2 ± 0.4	3.8 ± 0.1	477.0 ± 35.0	191.3 ± 16.9	2.49:1
*Cr*	Parent	I and II	4.2 ± 1.0	1.9 ± 0.2	1.3 ± 0.2	291.0 ± 30.1	110.5 ± 18.3	2.63:1
4	RIL	II	12.2 ± 0.3	5.0 ± 0.1	2.9 ± 0.1	599.3 ± 28.6	23.2 ± 3.3	25.78:1
9	RIL	II	6.9 ± 0.6	4.6 ± 0.2	2.3 ± 0.2	238.5 ± 31.1	107.8 ± 13.1	2.21:1
13	RIL	I	7.1 ± 0.2	4.9 ± 0.1	2.6 ± 0.4	291.4 ± 4.5	127.1 ± 19.3	2.29:1
27	RIL	I	7.1 ± 0.4	4.4 ± 0.3	2.6 ± 0.2	409.7 ± 59.7	83.8 ± 12.2	4.89:1
43	RIL	I	7.1 ± 0.1	4.5 ± 0.1	2.5 ± 0.4	300.1 ± 17.8	93.9 ± 18.0	3.19:1
49	RIL	II	7.0 ± 0.2	3.8 ± 0.2	2.7 ± 0.1	557.3 ± 39.5	99.7 ± 14.2	5.59:1
54	RIL	II	7.0 ± 0.1	4.4 ± 0.2	2.6 ± 0.2	359.7 ± 18.0	45.2 ± 2.2	7.95:1
55	RIL	I	6.4 ± 0.4	4.4 ± 0.1	2.8 ± 0.1	301.7 ± 50.0	26.9 ± 8.4	11.22:1
56	RIL	II	5.4 ± 0.7	3.5 ± 0.2	2.1 ± 0.3	418.6 ± 48.0	131.2 ± 6.3	3.19:1
90	RIL	II	11.6 ± 0.4	5.1 ± 0.2	3.6 ± 0.2	394.0 ± 16.8	46.0 ± 0.5	8.56:1
95	RIL	II	6.6 ± 0.2	4.2 ± 0.1	2.3 ± 0.1	179.6 ± 40.4	48.5 ± 11.8	3.70:1
139	RIL	I	8.3 ± 0.6	5.2 ± 0.1	2.9 ± 0.2	177.3 ± 60.5	37.5 ± 4.8	4.73:1
148	RIL	II	6.3 ± 0.2	4.4 ± 0.2	2.6 ± 0.2	300.6 ± 7.7	25.3 ± 6.8	11.90:1
156	RIL	I	7.8 ± 0.2	4.6 ± 0.3	2.6 ± 0.1	257.2 ± 46.3	39.3 ± 11.1	6.54:1
157	RIL	I	8.1 ± 0.4	5.0 ± 0.2	2.9 ± 0.2	274.3 ± 19.3	41.3 ± 16.6	6.64:1
185	RIL	I	13.9 ± 0.4	5.7 ± 0.2	3.4 ± 0.1	391.8 ± 17.1	93.3 ± 15.0	4.20:1
202	RIL	II	10.8 ± 0.7	5.1 ± 0.4	3.3 ± 0.2	396.5 ± 26.4	111.3 ± 18.9	3.56:1
207	RIL	II	7.2 ± 0.9	4.0 ± 0.1	3.0 ± 0.2	339.7 ± 33.0	49.1 ± 1.2	6.92:1
225	RIL	I	7.3 ± 0.3	4.7 ± 0.2	2.6 ± 0.4	331.0 ± 40.2	40.1 ± 4.0	8.25:1
231	RIL	I	8.1 ± 0.2	4.6 ± 0.1	3.2 ± 0.1	493.1 ± 3.5	108.1 ± 7.9	4.56:1

^a^Each group is a subset of 10 selected RILs and 2 parents tested together in the arena during foraging trials (see Materials and methods and Supplementary material).

**Fig. 3.**
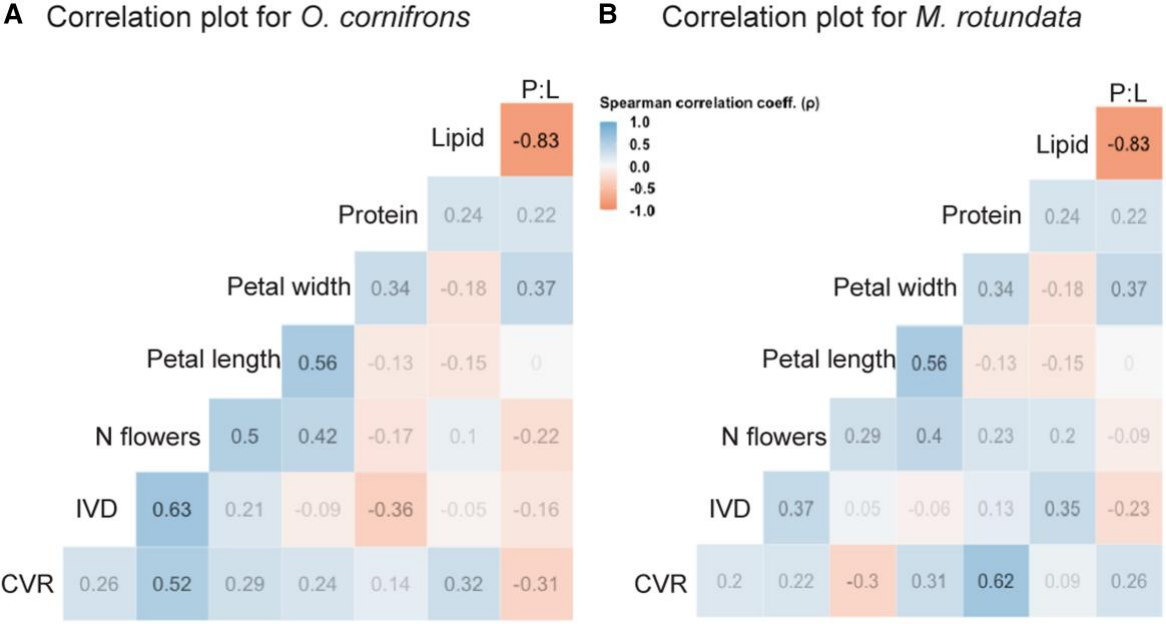
Spearman correlation plot among the factors considered in the analysis for two bee species, A) *O. cornifrons* and B) *M. rotundata*, foraging for the recombinant inbred plant lines of *Capsella* species produced by crossing obligate outcrosser and insect-pollinated *Cg* with the selfer *Cr* plant lines. Analysis was conducted on the average values for each variable factor. Values indicate pairwise Spearman correlation coefficients.

Among the floral traits, there was no significant correlation between any floral morphological traits (number of flowers, petal length or width) with any pollen nutritional factors (protein, lipid, and P:L) (Spearman-rank test *P* > 0.1; Fig. 3). However, a significant positive correlation between petal length and width (Spearman: *ρ* = 0.56, *P* = 0.01), and a strong negative correlation between lipid concentration and P:L ratios (*ρ* = −0.83, *P* = 1.54 × 10^−6^) were observed.

Thus, overall, crossing *Cg* and *Cr* uncoupled floral morphological and pollen nutritional traits, making it possible to evaluate bee responses to floral resource quantity and quality separately.

### Bees exhibit species-specific visitation patterns *Capsella* plant lines

Foraging preferences for each bee species was evaluated for 10 RILs and the parental lines in two groups, allowing 20 RILs to be evaluated. Bees were provided with sucrose solutions from artificial flowers prior to the foraging assay and both species of bees appear to foraging selectively for pollen from the flowers, since no bees were observed to probe the floral nectaries with their proboscises.

For *O. cornifrons*, a total of 552 and 445 single visits were recorded, respectively, for Group-I and Group-II plant lines by 66 active female bees through 18 indoor foraging arena trials. For *M. rotundata*, a total of 1,208 (Group-III) and 1,320 (Group-IV) visits were recorded with 112 active female bees in 20 trials. Both the cumulative visitation rates (CVR) and individual visitation duration (IVD) were recorded. While both bee species preferentially visited *Cg* vs. *Cr*, there was considerable variation in their visitation preferences to the RILs. For example, RIL 156 was the fourth most attractive RIL for *O. cornifrons*, but fourth least attractive for *M. rotundata* in terms of the CVR (see Fig. S1).

Figure 3 shows the correlation between factors (i.e. CVR, IVD number of flowers, petal length, petal width, protein concentration, lipid concentration, and P:L) considered for this study for each species of bees for 20 recombinant *Capsella* lines. Because the parental line *Cg* was highly attractive, neither of the parental lines was included in this analysis. However, see Fig. S2 for correlation plots using all 20 *Capsella* RILs and *Cg* and *Cr*.

For *O. cornifrons*, the CVR among RILs was significantly positively correlated with the number of flowers per inflorescence (*ρ* = 0.52, *P* = 0.02; Fig. 3a), while none of the other factors were significantly associated with CVR (*P* ≥ 0.18 for RILs, see Fig. 3a and *P* ≥ 0.22 for all plant lines, Fig. S2). Moreover, *O. cornifrons* females spent significantly more time during individual flower visits (individual visit duration [IVD]) on plant lines that presented a greater number of flowers (*ρ* = 0.63, *P* = 0.004, Fig. 3a).

In contrast, for *M. rotundata*, attraction to the RILs was positively correlated by the protein concentrations in pollen (*ρ* = 0.62, *P* = 0.004) and was unaffected by the number of flowers available for each plant line or their petal size (*P* ≥ 0.17) (see Fig. 3b). Leafcutting bees were generally attracted toward RILs with relatively greater proportions of protein to lipid (with RIL 90 that has 8.56:1 P:L being the most attractive line). However, the IVD of *M. rotundata* was not influenced by either the number of flowers, petal sizes or macronutrient concentrations in the pollen (*P* ≥ 0.11, Fig. 3b).

**Fig. 4.**
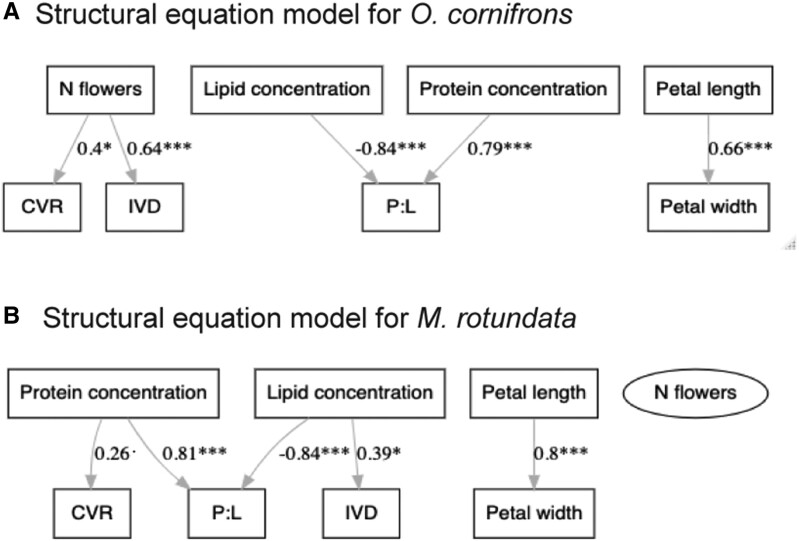
Structural equation model plots (using Lavaan) for the relationship between floral morphological and nutritional traits and bee visitation for two bee species A) *O. cornifrons* and B) *M. rotundata* for the 20 recombinant inbred plant lines of *Capsella* species produced by crossing obligate outcrosser and insect-pollinated *Cg* with the selfer *Cr* plant lines. Solid lines represent significant correlation coefficients with regression values, at *P* ≤ 0.001 (***), 0.001 < *P* ≤ 0.01(**), 0.01 < *P* < 0.05 (*), or *P* < 0.1(.) level. Best model fit was selected with ΔAIC = 0. Positive or negative sign of coefficients indicate direction of correlation between the factors. Oval shape indicates latent factors. See Supplementary material for full model estimates for each species.

Additionally, we conducted structural equation modeling (SEM) separately for each bee species to examine the most plausible direct and indirect links between factors by assessing conditional independence among indirectly linked variables (29). For *O. cornifrons*, the path analysis depicted consistent direct links between CVR or IVD and the number of flowers but no direct/indirect dependence on petal size or pollen macronutrients. In contrast, for *M. rotundata*, nutritional values (protein and lipid concentrations but not P:L), directly influenced bee foraging (either CVR or IVD), while there was no effect of flower number or petal size (see Fig. 4 for the best fit models with ΔAIC = 0; AIC, Akaike Information Criterion). Detailed results of SEM analyses were given in Supplementary material.

### Choice assays using artificial flowers demonstrate *M. rotundata* bees forage according to nutritional content

In previous studies in bumble bees, the foraging preferences for pollen P:L ratios were shown to correlate with their feeding preferences on artificial liquid diets with dissolved proteins and lipids (30, 31). Because liquid diets can be offered with different nutritional ratios but with similar visual cues, they were used as a feeding medium for our study (see Materials and methods).

In the lab-based foraging diet assays using artificial flowers with liquid diets, visits by *O. cornifrons* females were not influenced by the P:L ratios in diets ($\chi^{2}\;$ = 0.56, *P* = 0.46) or the color of artificial flowers ($\chi^{2}\;$ = 0.20, *P* = 0.65). In contrast, *M. rotundata* female bees preferred the 10:1 P:L diets vs. 1:1 P:L in sugar solution ($\chi^{2}\;$ = 5.0, *P* = 0.02), regardless of the flower color ($\chi^{2}$ = 0.02, *P* = 0.88) during the assay. The duration of their individual bee visits was not affected by the diet treatments or flower color for either bee species (*P* ≥ 0.46).

## Discussion

To the best of our knowledge, this is the first study to uncouple floral morphological and nutritional traits to analyze the foraging choices of two agriculturally important solitary bee species, *O. cornifrons* and *M. rotundata*. By using *Capsella* RILs, we were able to uncouple floral traits to precisely evaluate how these traits individually influenced foraging preferences. Our results clearly showed that the bees can exhibit species-specific foraging preferences in terms of perceived floral resource quantity (flower number) and floral resource quality (macronutrient content of the pollen).

Several studies have found that the dietary preferences and foraging behaviors of solitary bees can vary widely from species to species (32, 33). In our study, we observed that the foraging preferences of *O. cornifrons* are mainly driven by the number of flowers rather than the nutritional value of the *Capsella* pollen source (see Figs. 3 and 4). This suggests these bees might be adaptable to foraging on different plant species, but preferentially attracted to plants with large numbers of flowers. In the field, these bees typically forage on pollen from spring flowering trees, representing mass-blooming resources with large floral areas (34, 35). Holzschuh et al. (36) showed that mass flowering in agricultural habitats (with crops like oil seed rape belonging to Brassicaceae family) significantly attracts *Osmia bicornis*. However, it remains to be determined if the bees distinguish between trees or patches of different sizes and different flowering areas or if they simply are attracted to floral display sizes above a certain threshold. Vaudo et al. (34), through pollen metabarcoding, identified the phylogenetic origin and relative abundance of pollen in larval provisions of *O. cornifrons* bees in different landscapes. In their study, authors demonstrated that while *O. cornifrons* females frequently collect pollen from plants native to East Asia (their region of origin), they also collect pollen from plants/trees that belong to the same phylogenetic family or closely related plant groups depending on the landscape. In other words, *O. cornifrons* bees might venture beyond their typical pollen sources and collect pollen from related plants that they do not usually rely on to facilitate and diversify their foraging choices to support their survival and reproduction. In another study, it was found that dietary generalization is associated with significantly earlier phenology in *Osmia* bees, with generalists, such as *O. cornifrons*, being active (on average 11–14 days) earlier than specialists that rely on pollen from specific plant species (37). This behavior showcases the ecological flexibility of certain bee species. While they may have preferred pollen sources, they can adapt their foraging behaviors to changing environmental conditions or the availability of resources. Such adaptations could allow for spring-emerging solitary bees (such as *Osmia* bees) to overcome inherent risks of phenological asynchrony associated with early-spring phenology (38). In our study, by comparing *O. cornifrons* preferences to a suite of plant lines that vary in their floral traits, we demonstrate that these bees act as nutritional generalists and selectively forage based on floral area rather than nutritional content.

Bees that are exclusive dietary specialists rely on specific plant species as their primary food source. For example, *M. rotundata* is known to be a specialist pollinator on Fabaceae plants and is widely managed for alfalfa pollination due to its strong tendency to forage on this plant species for nest building and pollen collection (39). Specializing in plants that provide pollen with a specific nutritional content could be plausible for a summer emerging bee species (like *M. rotundata*) when floral resource availability and diversity are greater and/or more consistent than what is available to spring bees. Our study found that the *M. rotundata* visitation was significantly associated with the concentrations of macronutrients (particularly proteins) in *Capsella* pollen sources. Though this nutritional specialization is advantageous for commercial pollination purposes (such as for alfalfa crops), it can make bees more vulnerable to fluctuations in host-plant abundance, quality, or phenology (40, 41).

We used artificial flowers, which varied in the P:L ratio of the resources provided, to evaluate if these two bee species differentially prioritized floral resource nutritional quality in a different context. In this novel context, where individual bees were offered a choice between high (10:1) vs. low (1:1) P:L ratios in liquid diets, nutritional composition was again found to be the key driving force for feeding choices for *M. rotundata* but not for *O. cornifrons* bees. Similar studies demonstrated that *B. impatiens* bumble bees preferentially forage from liquid diets, isolated pollen, and flowering plants that provided a 5:1–10:1 P:L ratio (12, 42).

It is important to note that only pollen protein and lipid content were measured in our study, but because the pollination biology of *Cg* is very different than *Cr*, other aspects of pollen chemistry including volatile organic compounds that the flower emits can also influence bee foraging and may be responsible for nutritional preferences observed in our arena experiment. Bee preferences under field conditions could also be influenced by a variety of floral traits such as color and UV patterns, nectar quantity, and quality, etc. However, the foraging choices of two bee species nonetheless correlated with protein and lipid concentrations, and results from the arena studies were supported by the artificial feeders’ experiment, indicating that these factors are behaviorally relevant (43). Moreover, though there is no evidence for social learning of foraging preference in our study species, our experimental design did not account for that, and future studies should be conducted using controlled arenas to examine this possibility further. Considering all these additional floral traits and behaviors and conducting experiments to understand species-specific bee preferences is necessary to understand the co-evolution of plant–pollinator interactions and develop habitat management and restoration schemes that can most effectively support pollinator communities and their essential pollination services.

Understanding plant–pollinator dynamics can inform plant breeding programs and landscape design practices, enabling the creation of habitats that are ecologically beneficial and attract and support a diverse community of pollinators. Moreover, unraveling the intricate relationships between plant traits and their specific bee pollinators can provide insights into how these systems evolve, at a species and community level.

## Materials and methods

All experiments were conducted in a laboratory facility at the Huck Institutes of Life Sciences, Pennsylvania State University, United States.

### Bee species

Two commercially managed solitary bee species in the family Megachilidae, *O. cornifrons* and *M. rotundata* were selected for the study. Emerged bees from purchased cocoons were maintained in a walk-in environmental chamber set at 23 ± 1 °C, 65 ± 5% relative humidity (RH) and 16:8 h light:dark (L:D) regime, and fed ad libitum with 30% w/w sucrose solution (see Supplementary material).

### *Capsella* plant lines

Several recombinant interspecific plant lines were generated by crossing *Cg* and *Cr* by Sicard lab at Swedish University of Agricultural Sciences, Sweden (as described in Sicard et al. (28)). Out of 20 RILs with relatively high degree of variation in phenotypic traits and good rate of germination were selected for the study along with their parental plant lines, *Cg* and *Cr*. Details of selected plant lines and their morphological and physiological characteristics are presented in Table 1. Seeds from each *Capsella* plant line were germinated in the environmental chamber (16:8 h L:D, 23 ± 1 °C temperature and 65 ± 5% RH) and later grown in the greenhouse under controlled conditions. Detailed procedure for plant germination and growth was described in Supplementary material.

### Pollen nutritional content of *Capsella* plant lines

In May to June 2022, hundreds of fresh flowers from four plants of each plant line were collected and dried in the incubator at 36 °C for 24 h. As the flowers from *Capsella* spp. are relatively small, pollen was pooled from all collected flowers to get a sufficient quantity for sample analysis (>5 mg). We used a protocol modified from Erickson et al. (18) for collecting pollen from small flowers and the pollen was stored at −20 °C until analysis. Total protein and lipid contents in pollen were analyzed using protocols detailed previously in Erickson et al. (18) and Vaudo et al. (12). See Supplementary material document for details on pollen collection methodology and nutritional measurements. Average protein and lipid concentrations were determined in three subsamples of pollen (mean ± SE µg/mg); Table 1.

### Lab-based foraging arena assays

Pollinator foraging preferences were assessed in the laboratory during 2022 April 19 to May 4 for *O. cornifrons* and 2022 June 6 to 17 for *M. rotundata*. Figure 1 shows the schematic representation of the foraging assay in the flight arena and bee visitation.

On the day of foraging assay, fresh inflorescences from each plant line were collected in the morning between 8 and 10 AM and brought to the lab. Each inflorescence was cut at 9 cm (while retaining the seed pods to maintain visual cues as observed in the field) and placed in vertical green colored pointed floral tubes (Royal Imports, NY, USA) filled with water (see representative figure in Supplementary material). The number of flowers per inflorescence were counted and used as a representation of “floral resource quantity.” The floral tubes were fixed to the back wall of the custom-built flight arena in an array (see Fig. 1 and Supplementary material), and plant lines were randomly assigned to the array of tubes for each foraging trial. Each arena consists of 12 holders to keep flowers (see Supplementary material), so the experiment was conducted in two groups, each with flowers from 10 different RILs and 2 parental plant lines. Mated females (5 bees for *O. cornifrons* or 10 for *M. rotundata*) were uniquely paint-marked for individual identification and released into the arena. When a bee touched the reproductive/flowering part of the inflorescence including petals (excluding stem/plant holder) in the arena, it was counted as a “visit.” Note that in some social bee species, foragers will preferentially forage on flowers that have been visited by a conspecific, and/or will avoid flowers that have scent marks left from a conspecific. Our study design did not control for these possible interactions, but these behaviors have not been described for our two focal species. Bee visits and time spent on the flowers of each plant line were continuously recorded for 1 h using MultiTimer application on a mobile phone. Though it is not entirely possible to differentiate pollen vs. nectar visits, bees were observed to mostly foraged for collecting pollen (waggling their abdomen onto the flower) and were not observed to probe the flower with their proboscis, though they were observed to extend their proboscises to drink water from the plant tube. The trial duration was standardized to 1 h to get enough visits while ensuring bees were able to explore flowers with intact pollen. After 1 h of observations, bees were removed from the arena and supplied with water. To check if the bees developed learned preferences for plant lines, they were rereleased after 90 min and assayed on fresh flowers (in the same array configuration) in the arena. A total of 20 foraging trials (10 trials per group of plant lines) were conducted for each species of bees. Only bees which are actively visiting with at least 3 visits per trial were considered for the data analysis (at least two *O. cornifrons* or four *M. rotundata* were active in each foraging trial). Data analysis using the number of active bees per trial yielded similar results as analysis with number of released bees in the arena for each species, suggesting no density-dependent shifts in selectivity for foraging of these bees.

Bee foraging preferences among the plant lines were compared directly by creating metrics at both the community and individual bee level, as in Vaudo et al. (16). Two behavioral metrics were used to assess the bees’ visitation in the arena; CVR defined by the total number of times a plant line was visited in an hour by all bees in the arena (visits/h/5 bees for *O. cornifrons* and visits/h/10 bees for *M. rotundata*), and IVD as the time spent by a single bee on each plant line (seconds/visit/bee) for pollen. These metrics help to reduce the variation in the data caused by the number of bees in the environment.

### Diet-based artificial flower choice assays

To understand whether bees exclusively forage based on P:L ratios in food, a two-choice-based lab assay was performed using artificial diets at 1:1 and 10:1 P:L in 0.5 mol/L sucrose solution for each species (*O. cornifrons* and *M. rotundata*) (see Supplementary material for details). Liquid diets were used because they are easy for the bees to access, ingest and ensure that their visits were solely for food collection. Each foraging trial, experimental cage was equipped with four Eppendorf tubes of each diet (1:1 and 10:1 P:L) and fixed with either pink or blue paper flowers to facilitate bees’ learning for diet treatments. The color of flowers for diet treatments was changed between trials. For the foraging trial, five individually paint-marked bees were released into the experimental cage fixed with feeding tubes (4 for 1:1 and 4 for 10:1 P:L) and their visits and duration were recorded for 30 min. A total of 15 trials each for *O. cornifrons* and *M. rotundata* were conducted to get 15 unique bees with at least 3 visits for each species. Inactive bees with <3 visits were excluded from the analysis.

### Statistical analysis

All statistical tests and plotting were performed in R version 3.5.0 (R Foundation for Statistical Computing, Vienna, Austria). To describe the variation in nutritional distribution across plant lines, the concentrations of total lipids (mean ± SE) were plotted against total protein concentrations (mean ± SE). In addition, the overall P:L ratios were explained by drawing linear “rails” alongside a range of specific nutrient concentrations (Fig. 2).

Nonparametric models were used for statistical testing on *z*-transformed multivariate data using “scale” function in R (44). Spearman correlations were used to assess the relationship between floral morphological traits (number of flowers, petal length, and width) or nutritional metrics (protein concentration, lipid concentration, and P:L) and bee visitation (CVR and IVD) on scaled data for each bee species. Because the pollinator-dependent parental line *Cg* received significantly highest number of visitations than any RIL (except RIL90 or RIL185) for both bee species, to understand bees’ preferences within RILs and eliminate *Cg* bias, separate correlation plots were modeled only for RILs by excluding data for both parental plant lines (*Cg* and *Cr*), though the plots using all of the plant lines are presented in the Supplementary material.

SEM was used to analyze the covariance and causal relationships among floral morphological, nutritional factors, and bee visitation in the arena individually for each species. Based on correlation plots, we hypothesized that, either floral morphological or nutritional traits influence CVR and/or IVD directly but also indirectly by linking with each other. In addition, some floral or nutritional factors could be intra-dependent with each other (i.e. P:L and petal size). Therefore, we preselected possible path combinations, by analyzing the four response variables of our path model (i.e. CVR, IVD, petal width, and P:L) for each bee species separately. CVR and IVD were analyzed with all explaining variables, while petal width only for petal length, and P:L for both protein and lipid concentrations. Normalized and scaled data for each variable was used to achieve more uniform residual variance measures with models being estimated using the full information maximum likelihood estimator. All SEMs were built and tested using the R package “lavaan.” The best supported path model was selected by comparing AICs (for which ΔAIC = 0).

To understand the foraging choices exclusively based on nutrition, observed bee visits during artificial diet assays were analyzed using χ^2^ goodness-of-fit test assuming equal proportions of 1:1 and 10:1 P:L diet treatments, and the duration of visits were analyzed using student *t* test.

## Supplementary Material

pgae443_Supplementary_Data

## Data Availability

Data for these studies are available through Penn State University's ScholarSphere database doi: 10.26207/1vyw-wd86.
