# Supplementary material for: Foraging bee species differentially prioritize quantity and quality of floral rewards
Source: PNAS Nexus. 2024 Oct 7;3(10):pgae443. doi: 10.1093/pnasnexus/pgae443 (PMC11477986; doi:10.1093/pnasnexus/pgae443)
Supplement: pgae443_Supplementary_Data [file pgae443_supplementary_data.docx]

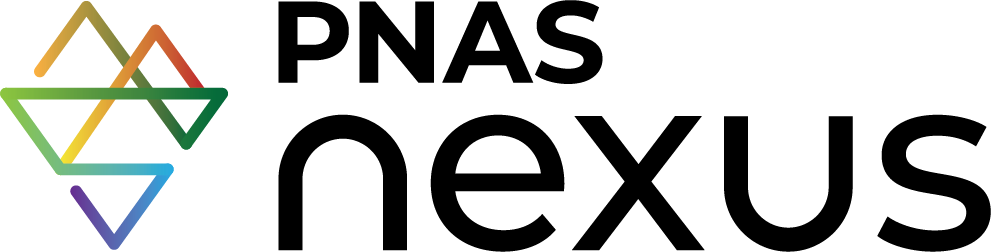


**Supporting Information for**

Foraging bee species differentially prioritize quantity and quality of floral rewards.

Jaya Sravanthi Mokkapati^1*^, Michael Hill^1^, Natalie Boyle^1^, Pierre Ouvrard^2^, Adrien Sicard^2^, Christina M. Grozinger^1^

Author for correspondence: Jaya Sravanthi Mokkapati - JM

Email: [jayasravanthimokkapati@gmail.com](mailto:jayasravanthimokkapati@gmail.com)

**This PDF file includes:**

Supporting text and results

Figure S1, S2

Supporting Information Text

**Rearing and maintenance of bees**

Only mated female bees were used in the study, as these are the bees most likely to be actively collecting pollen to bring back to their nest to create brood provisions for their offspring [1]. Cocoons of both bee species were purchased from a commercial supplier (Watts Solitary Bees, Bothell, WA, USA). Cocoons were stored as loose cells in dark conditions at 4^o^C until use, whereby adults were encouraged to emerge via established management protocols (for *Osmia spp.* see Bosch and Kemp, (1); for *M. rotundata,* see Baird and Bitner, (2))*.*

For *O. cornifrons*, in April 2022, ca. 100 cocoons containing mixed males and females were placed in a petri dish and introduced to emergence/maintenance cages (30×30×30 cm) constructed from a combination of acrylic sheets and 1/16 in (0.16 cm) hardware cloth. Emergence cages were kept in a walk-in environmental chamber set at 23±1°C, 65±5% relative humidity (RH) and 16:8 hours light:dark (L:D) regime. Twice daily (morning and evening), bees were observed for emergence and mating, and were fed *ad libitum* with 33% sucrose solution. Emerged females were held in the cages for at least three days prior to introduction into foraging arenas to allow time for mating to occur.

For *M. rotundata*, in May 2022, loose cocoons were placed in petri dishes (4 g per dish) and acclimatized to a gradual increase in temperature at 23±1^o^C for 8 hours followed by 29±1°C in incubators with 65±5% RH. Petri dishes of cocoons with fungal growth or parasitic wasp activity were removed as observed. For parasite control in the developing cells, on Day 7 of incubation, a pesticide strip of dichlorvos (Vapona™) was placed in the incubator (3/4^th^ strip per 1000 cubic ft (or 28.3 m^3^) of incubator space, per Baird and Bitner, [3]) and was removed on Day 13, after which we thoroughly aerated the incubator with additional fans. Once males started to emerge, dishes were observed twice daily, and individual adults were moved and maintained in cages (with equal number of males and females per cage) in an environmental chamber with *ad libitum* access to 33% sucrose solution, similar to *O. cornifrons* above. Emerged females were maintained in the cages for at least three days prior to introduction to the foraging arenas.

**Plant germination and growth**

Seeds from each *Capsella* plant line were germinated in long disposable black trays on potting media (Sunshine® Mix #4 professional growing soil from Sun Go Horticulture, USA) with supplemental lighting in the climatic chamber (16:8 h L:D, 23±1°C temperature and 65±5% RH). To promote germination, trays with seeds were first cold vernalized for 4 – 6 days at 4^o^C. If the seeds did not germinate with 48 hours, the media was supplemented with 50 μM gibberellic acid (GA) (all *Cg* plants required GA supplementation). Seedlings were later transferred to 700 mL pots with potting media and maintained in the greenhouse under 16:8 hours L:D, 23±1°C temperature, 65±5% RH and with greater light intensity (from the Sun) during the day. Plants were grown in two batches for foraging assays with each bee species: from February 2022 for *O. cornifrons* and from April 2022 for *M. rotundata* foraging assays. On 23 May 2022, plants were treated against aphids with a pymetrozine insecticide Endeavor®, which is known to be relatively non-toxic to pollinators [4]. Foraging experiments were performed at least 14 days following the application of insecticide treatments to minimize any chemical cues associated with the chemical by allowing time for its natural degradation. The dimensions of petal size for each plant line were measured using a digital caliper and petal area through images (by ImageJ) in triplicates and averaging across three different flowers.

**Procedure for pollen extraction from *Capsella* flowers**

To remove pollen from anthers, pooled flower samples were transferred to modified spin filter tubes fitted with 100-micron stainless steel mesh. Samples were then agitated using a bead mill homogenizer (OMNI International Lenesaw, GA) at 4.0 m/s in four 30 second increments to extract pollen grains from the samples. Samples were centrifuged for five minutes at 13000 RPM and lyophilized (Labconco FreeZone 1177030, Kansas City MO) for three hours at -80°C to remove all moisture. Pollen samples were then weighed into six separate vials (0.5 mg – 1.3 mg) using a microbalance (Mettler Toledo XPE26) and stored at -20^o^C to be used for protein and lipid analysis later.

**Procedure for nutritional measurements in pollen**

Total protein and lipid contents in pollen were analyzed using protocols detailed previously in Erickson et al. [5]and Vaudo et al. [6]. Briefly, a Bradford assay was used to measure the total protein concentration from 0.7±0.3 mg of pollen, using a standard curve created with bovine serum albumin (BSA, Sigma-Aldrich, USA) (as in [6]). Total lipid concentrations were measured in 0.9±0.4 mg of pollen using an assay modified from Van Handel and Day, [7] while adjusting the amount of Vanillin reagent used based on sample weight. The amount of extracted lipids was determined by reading absorbance at 525 nm using canola oil as the standard. All measurements were recorded in triplicate using SpectraMax® 190 Microplate Reader (Molecular Devices, San Jose, CA, USA).

**Foraging trials using *Capsella* plant lines**

Experiments were conducted in two custom-built flight arenas (90x54x46 cm), where one side is covered with plexiglass to allow for observation (as in [5]). To ensure bees could fly freely in search of food, before each planned foraging assay, female bees were acclimatized to the flight arenas for 1-2 days by providing *ad libitum* sucrose solution in several small dishes. Each arena consists of 12 holders (4×3) to keep flowers, so the experiment was conducted in two groups, each with flowers from 10 different RILs and 2 parental plant lines.

**Artificial flower diet-choice Assay**

Newly emerged mated females were fed *ad libitum* with 0.5 mol/L sucrose solution in small cages (25x20x20 cm - closed with mesh on all sides except base). Food was provided in eight 1.5 mL Eppendorf tubes fixed with black colored paper flowers to train the bees to locate food and acclimatize the cages. Two treatment diets at 1:1 and 10:1 P:L were prepared following the protocol by Vaudo et al. [6], using casein sodium salt from bovine milk (Sigma-Aldrich) for protein, and 100% sunflower lecithin (Micro Ingredients USDA organic supplements, USA) for lipids (>94% fat), in 0.5 mol l-1 sucrose (Sigma-Aldrich, St. Louis, MO, USA) solution. Sunflower lecithin is an emulsifier that can be dissolved in sucrose, and hence used for preparing liquid diets.

**Statistical Analysis**

Bee visitation parameters such as Cumulative visitation rates (CVR) and Individual Visit Duration (IVD) for each bee species were correlated with all possible factors on scaled data using Spearman correlation analysis (check the results below). Descriptive factors such as plant ID, arena, observer, time, trial, position, and angle of flower in the arena were randomized and/or non-significant in preliminary generalized linear model analysis (P > 0.05), thus were not considered for further analyses.

Next, path analysis was carried out using structured equation models considering four response variables (CVR, IVD, petal width, P:L) with corresponding explaining variables for each bee species (on normalized and scaled data) (see manuscript). Results for the best fit model (with least AIC values) were presented below (see Figure 4 in the manuscript to visualize models).

***
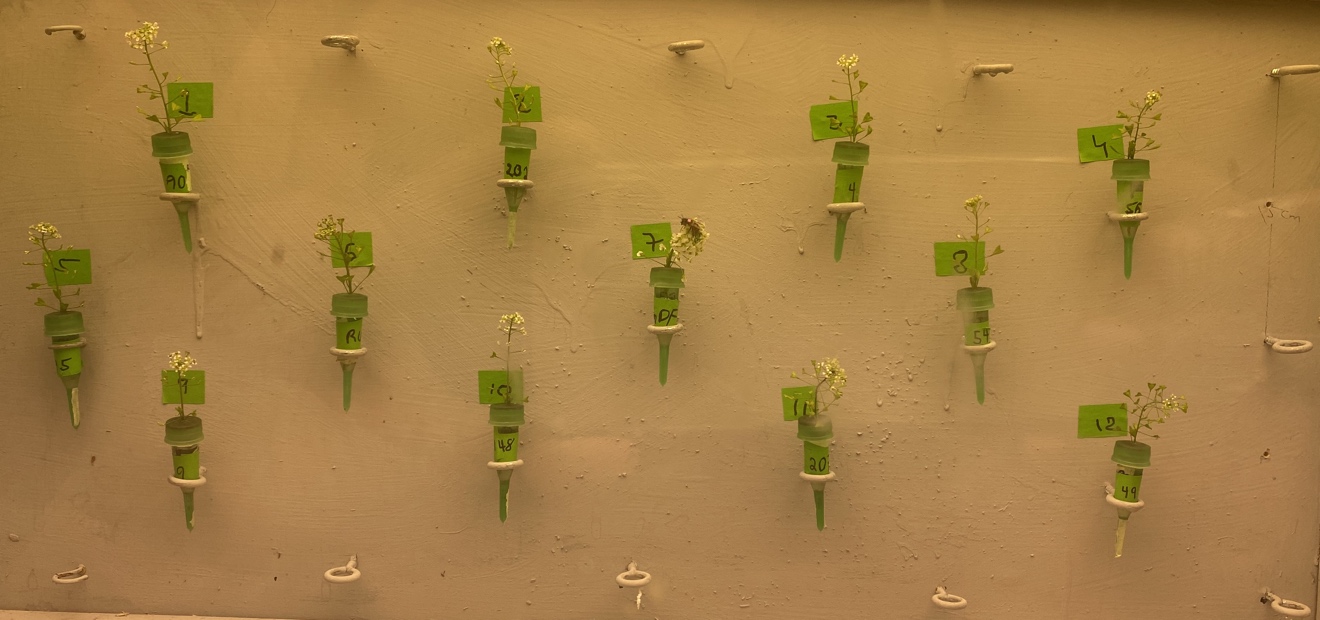
***

Representative image of foraging arena containing *Capsella* flowers - in each trial, the arena was equipped with 12 different inflorescences from 10 *Capsella* recombinant plant lines and their 2 parents (*C. grandiflora* and *C. rubella*).

**SI References**

1. Raw A. 1972 The biology of the solitary bee Osmia rufa (L.) (Megachilidae). *Transactions of the Royal Entomological Society of London* **124**, 213–229. (doi:10.1111/j.1365-2311.1972.tb00364.x)

2. Bosch J, William P. Kemp. 2001 *How to manage the blue orchard bee*. Sustainable Agriculture Network, Beltsville, MD.

3. Baird CR, R. M. Bitner. 1991 Loose cell management of leafcutting bees. In *In Alfalfa Seed Production and Pest Management*, Western Regional Extension Publications.

4. Jansen JP, Defrance T, Warnier AM. 2011 Side effects of flonicamide and pymetrozine on five aphid natural enemy species. *BioControl* **56**, 759–770. (doi:10.1007/s10526-011-9342-1)

5. Erickson E, Junker RR, Ali JG, McCartney N, Patch HM, Grozinger CM. 2022 Complex floral traits shape pollinator attraction to ornamental plants. *Ann Bot* **130**, 561–577. (doi:10.1093/aob/mcac082)

6. Vaudo AD *et al.* 2020 Pollen protein: Lipid macronutrient ratios may guide broad patterns of bee species floral preferences. *Insects* **11**. (doi:10.3390/insects11020132)

7. Lau P, Grebenok RJ, Rangel J, Behmer ST. 2022 Assessing pollen nutrient content: A unifying approach for the study of bee nutritional ecology. *Philosophical Transactions of the Royal Society B: Biological Sciences* **377**. (doi:10.1098/rstb.2021.0510)

**Figure S1.** Cumulative visitation rates of (a) *Osmia cornifrons* and (b) *Megachile rotundata* bees for the recombinant inbred plant lines of *Capsella* produced by crossing pollinator-dependent *C. grandiflora* (*Cg*) and self-reproducing *C. rubella* (*Cr*) (indicated by upward arrows). A total of 38 trials for *O. cornifrons* and 40 trials for *M. rotundata* were conducted using individually paint-marked mated females (5 bees/trial for *O. cornifrons* and 10 bees/trial for *M. rotundata*).

**Figure S2.** Spearman correlation plots between the factors considered in the analysis for two bee species foraging for the twenty recombinant inbred plant lines of *Capsella* species produced by crossing obligate outcrosser and insect-pollinated *C. grandiflora* (*Cg*) with the selfer *C. rubella* (*Cr*) plant lines (including parental lines). Analysis was conducted on the average values for each variable factor. Values indicate pairwise Spearman correlation coefficients with a gradient color between blue (positive correlation) and orange (negative correlation).

***Results of the structural equation model (SEM) for the factors influencing foraging activity of female O. cornifrons bees on the Capsella sps. plant lines. Data included 20 Cg x Cr recombinant inbred lines (RILs) excluding their parents Cg and Cr.***

| lavaan 0.6-18 ended normally after 19 iterations | |
| --- | --- |
|  |  |
| Estimator | ML |
| Optimization method | NLMINB |
| Number of model parameters | 15 |
| Number of observations | 20 |
|  |  |
| Model Test User Model: |  |
| Test statistic | 16.385 |
| Degrees of freedom | 11 |
| P-value (Chi-square) | 0.127 |
|  |  |
| Model Test Baseline Model: |  |
| Test statistic | 91.406 |
| Degrees of freedom | 22 |
| P-value | 0 |
|  |  |
| User Model versus Baseline Model: |  |
| Comparative Fit Index (CFI) | 0.922 |
| Tucker-Lewis Index (TLI) | 0.845 |
|  |  |
| Loglikelihood and Information Criteria: |  |
| Loglikelihood user model (H0) | -73.953 |
| Loglikelihood unrestricted model (H1) | -65.760 |
|  |  |
| Akaike (AIC) | 177.906 |
| Bayesian (BIC) | 192.842 |
| Sample-size adjusted Bayesian (SABIC) | 146.600 |
|  |  |
| Root Mean Square Error of Approximation: |  |
| RMSEA | 0.156 |
| 90 Percent confidence interval - lower | 0.000 |
| 90 Percent confidence interval - upper | 0.304 |
| P-value H_0: RMSEA <= 0.050 | 0.156 |
| P-value H_0: RMSEA >= 0.080 | 0.795 |
|  |  |
| Standardized Root Mean Square Residual: |  |
| SRMR | 0.137 |
|  |  |
| Parameter Estimates: |  |
| Standard errors | Standard |
| Information | Expected |
| Information saturated (h1) model | Structured |

| Regressions: |  |  |  |  |  |  |
| --- | --- | --- | --- | --- | --- | --- |
|  | Estimate | Std.Err | z-value | P(>\|z\|) | Std.lv | Std.all |
| CVR_Osmia ~ |  |  |  |  |  |  |
| N_flowers_Osmia | 0.40 | 0.20 | 2.04 | 0.041 | 0.40 | 0.41 |
| P:L ~ |  |  |  |  |  |  |
| lipid_conc | -0.84 | 0.07 | -11.29 | 0.000 | -0.84 | -0.75 |
| protein_conc | 0.78 | 0.07 | 10.65 | 0.000 | 0.78 | 0.70 |
| IVD_Osmia ~ |  |  |  |  |  |  |
| N_flowers_Osmia | 0.64 | 0.15 | 4.22 | 0.000 | 0.64 | 0.64 |
| petal_width ~ |  |  |  |  |  |  |
| petal_length | 0.66 | 0.11 | 5.99 | 0.000 | 0.66 | 0.65 |
|  |  |  |  |  |  |  |
| Covariances: |  |  |  |  |  |  |
|  | Estimate | Std.Err | z-value | P(>\|z\|) | Std.lv | Std.all |
| .CVR_Osmia ~~ |  |  |  |  |  |  |
| .P:L | -0.083 | 0.095 | -0.870 | 0.385 | -0.083 | -0.198 |
| .IVD_Osmia | -0.065 | 0.148 | -0.441 | 0.659 | -0.065 | -0.099 |
| .petal_width | 0.089 | 0.150 | 0.590 | 0.555 | 0.089 | 0.133 |
| .P:L ~~ |  |  |  |  |  |  |
| .IVD_Osmia | 0.026 | 0.080 | 0.328 | 0.743 | 0.026 | 0.074 |
| .petal_width | -0.251 | 0.099 | -2.550 | 0.011 | -0.251 | -0.694 |
|  |  |  |  |  |  |  |
| .IVD_Osmia ~ |  |  |  |  |  |  |
| .petal_width | -0.260 | 0.141 | -1.848 | 0.065 | -0.260 | -0.454 |
|  |  |  |  |  |  |  |
| Variances: |  |  |  |  |  |  |
|  | Estimate | Std.Err | z-value | P(>\|z\|) | Std.lv | Std.all |
| .CVR_Osmia | 0.768 | 0.243 | 3.162 | 0.002 | 0.768 | 0.833 |
| .P:L | 0.227 | 0.072 | 3.162 | 0.002 | 0.227 | 0.188 |
| .IVD_Osmia | 0.567 | 0.179 | 3.162 | 0.002 | 0.567 | 0.595 |
| .N_flowers_Osmia | 0.578 | 0.183 | 3.162 | 0.002 | 0.578 | 0.582 |
|  |  |  |  |  |  |  |
| R-Square: |  |  |  |  |  |  |
|  | Estimate |  |  |  |  |  |
| CVR_Osmia | 0.167 |  |  |  |  |  |
| P:L | 0.812 |  |  |  |  |  |
| IVD_Osmia | 0.405 |  |  |  |  |  |
| petal_width | 0.418 |  |  |  |  |  |

***Results of the structural equation model (SEM) for the factors influencing foraging activity of female M. rotundata bees on the Capsella sps. plant lines. Data included 20 Cg x Cr recombinant inbred lines (RILs) excluding their parents Cg and Cr.***

| lavaan 0.6-18 ended normally after 19 iterations | |
| --- | --- |
|  |  |
| Estimator | ML |
| Optimization method | NLMINB |
| Number of model parameters | 15 |
| Number of observations | 20 |
|  |  |
| Model Test User Model: |  |
| Test statistic | 9.313 |
| Degrees of freedom | 7 |
| P-value (Chi-square) | 0.231 |
|  |  |
| Model Test Baseline Model: |  |
| Test statistic | 82.89 |
| Degrees of freedom | 18 |
| P-value | 0.00 |
|  |  |
| User Model versus Baseline Model: |  |
| Comparative Fit Index (CFI) | 0.964 |
| Tucker-Lewis Index (TLI) | 0.908 |
|  |  |
| Loglikelihood and Information Criteria: |  |
| Loglikelihood user model (H0) | -74.675 |
| Loglikelihood unrestricted model (H1) | -70.019 |
|  |  |
| Akaike (AIC) | 179.351 |
| Bayesian (BIC) | 194.287 |
| Sample-size adjusted Bayesian (SABIC) | 148.045 |
|  |  |
| Root Mean Square Error of Approximation: |  |
| RMSEA | 0.129 |
| 90 Percent confidence interval - lower | 0.000 |
| 90 Percent confidence interval - upper | 0.322 |
| P-value H_0: RMSEA <= 0.050 | 0.262 |
| P-value H_0: RMSEA >= 0.080 | 0.689 |
|  |  |
| Standardized Root Mean Square Residual: |  |
| SRMR | 0.169 |
|  |  |
| Parameter Estimates: |  |
| Standard errors | Standard |
| Information | Expected |
| Information saturated (h1) model | Structured |

| Regressions: |  |  |  |  |  |  |
| --- | --- | --- | --- | --- | --- | --- |
|  | Estimate | Std.Err | z-value | P(>\|z\|) | Std.lv | Std.all |
| CVR_ALCB ~ |  |  |  |  |  |  |
| protein_conc | 0.26 | 0.15 | 1.81 | 0.070 | 0.26 | 0.29 |
| P:L ~ |  |  |  |  |  |  |
| lipid_conc | -0.84 | 0.08 | -10.80 | 0.000 | -0.84 | -0.74 |
| protein_conc | 0.81 | 0.08 | 10.42 | 0.000 | 0.81 | 0.71 |
| IVD_ALCB ~ |  |  |  |  |  |  |
| lipid_conc | 0.39 | 0.20 | 1.99 | 0.046 | 0.39 | 0.39 |
| petal_width ~ |  |  |  |  |  |  |
| petal_length | 0.80 | 0.11 | 7.14 | 0.000 | 0.80 | 0.71 |
|  |  |  |  |  |  |  |
| Covariances: |  |  |  |  |  |  |
|  | Estimate | Std.Err | z-value | P(>\|z\|) | Std.lv | Std.all |
| .CVR_ALCB ~~ |  |  |  |  |  |  |
| .P:L | -0.24 | 0.11 | -2.19 | 0.028 | -0.24 | -0.56 |
| .IVD_ALCB | 0.25 | 0.18 | 1.36 | 0.175 | 0.25 | 0.32 |
| .petal_width | 0.43 | 0.18 | 2.41 | 0.016 | 0.43 | 0.64 |
| .P:L ~~ |  |  |  |  |  |  |
| .IVD_ALCB | -0.11 | 0.10 | -1.06 | 0.290 | -0.11 | -0.24 |
| .petal_width | -0.27 | 0.10 | -2.60 | 0.009 | -0.27 | -0.72 |
| .IVD_ALCB ~~ |  |  |  |  |  |  |
| .petal_width | 0.09 | 0.16 | 0.57 | 0.570 | 0.09 | 0.13 |
|  |  |  |  |  |  |  |
| Variances: |  |  |  |  |  |  |
|  | Estimate | Std.Err | z-value | P(>\|z\|) | Std.lv | Std.all |
| .CVR_ALCB | 0.750 | 0.237 | 3.162 | 0.002 | 0.750 | 0.919 |
| .P:L | 0.233 | 0.074 | 3.162 | 0.002 | 0.233 | 0.188 |
| .IVD_ALCB | 0.816 | 0.258 | 3.162 | 0.002 | 0.816 | 0.849 |
| .petal_width | 0.606 | 0.192 | 3.162 | 0.002 | 0.606 | 0.498 |
|  |  |  |  |  |  |  |
| R-Square: |  |  |  |  |  |  |
|  | Estimate |  |  |  |  |  |
| CVR_ALCB | 0.081 |  |  |  |  |  |
| P:L | 0.812 |  |  |  |  |  |
| IVD_ALCB | 0.151 |  |  |  |  |  |
| .petal_width | 0.502 |  |  |  |  |  |
